# Saunders’ Question Compends, 8 and 9

**Published:** 1891-02

**Authors:** 


Saunders' Question Compends, 8 and 9. Essentials of Practice of Medicine, Arranged in form of Questions and Answers. Prepared Especially for Students of Medicine. By Henry Morris, M. D., Late Demonstrator Jefferson Medical College, Philadelphia, Visiting Physician to St. Joseph's Hospital, Etc. With a Complete Appendix on the Examination of Urine by Lawrence Wolff, M. D., Demonstrator of Chemistry, Jefferson Medical College. W. B. Saunders, 913 Walnut street, Philadelphia, 1890.

This work was written "especially for Students of Medicine," as stated in the preface. It is presented in form of questions and answers, thereby calling attention to the most important leading facts, the knowledge of which is not only desirable, but indispensable to an acquaintance with the Essentials of Medicines. Information is rendered more palatable when tendered in such manner as will secure attention and increase study. This little book is all that it pretends to be, and we cheerfully commend it to medical students desiring systematic knowledge in the department of medical practice.

The Appendix is worth the price of the book. The analysis of urine is made easy to all. Dr. Wolff appears to be master of the subject. A copy of this Appendix ought to be placed in the hands of every medical examiner for life insurance, who is not an expert in urinary analysis. A. W. G.

